# Diagnostic performance of quantitative Ga-SPECT/CT for patients with lower-limb osteomyelitis

**DOI:** 10.1186/s41824-022-00148-z

**Published:** 2022-12-01

**Authors:** Yoshito Nishikawa, Yoshimitsu Fukushima, Sonoko Kirinoki, Gen Takagi, Masaya Suda, Toshio Maki, Shinichiro Kumita

**Affiliations:** 1 Department of Radiology, Nippon Medical School, 1-1-5, Sendagi, Bunkydo-ku, Tokyo, 113-8603 Japan; 2 Department of Cardiovascular Medicine, Nippon Medical School, Tokyo, Japan

**Keywords:** SPECT/CT, Quantitative analysis, Osteomyelitis, Diagnostic performance, Prognostic value

## Abstract

**Background:**

Patients with lower-limb osteomyelitis (LLOM) may experience major adverse events, such as lower-leg amputations or death; therefore, early diagnosis and risk stratification are essential to improve outcomes. Ga-scintigraphy is commonly used for diagnosing inflammatory diseases. Although the diagnostic performance of planar and SPECT imaging for localized lesions is limited, SPECT/CT, which simultaneously acquires functional and anatomical definition, has resulted in significant improvements to diagnostic confidence. While quantitative Ga-SPECT/CT is an emerging approach to improve diagnoses, its diagnostic performance has not been sufficiently evaluated to date. Therefore, this study aimed to evaluate the diagnostic performance of Ga-SPECT/CT with quantitative analyses for patients with LLOM.

**Methods:**

A total of 103 consecutive patients suspected of LLOM between April 2012 and October 2016 were analyzed. All patients underwent Ga-scintigraphy with SPECT/CT imaging. Findings were assessed visually, with higher than background accumulation considered positive, and quantitatively, using Ga-SPECT/CT images to calculate the lesion-to-background ratio (LBR), the maximum standardized uptake value (SUVmax), and total lesion uptake (TLU). Diagnoses were confirmed using pathological examinations and patient outcomes, and diagnostic performances of planar, SPECT, and SPECT/CT images were compared. To evaluate prognostic performance, all patients were observed for 5 years for occurrences of major adverse events (MAE), defined as recurrence of osteomyelitis, major leg amputation, or fatal event. Multivariate Cox regression was performed to evaluate outcome factors.

**Results:**

The overall diagnoses indicated that 54 out of 103 patients had LLOM. LBR, SUVmax, and TLU were significantly higher in patients with LLOM (12.23 vs. 1.00, 4.85 vs. 1.34, and 68.77 vs. 8.63, respectively; *p* < 0.001). Sensitivity and specificity were 91% and 96% for SPECT/CT with LBR, 89% and 94% for SPECT/CT with SUVmax, and 91% and 92% for SPECT/CT with TLU, respectively. MAE occurred in 23 of 54 LLOM patients (43%). TLU was found to be an independent prognostic factor (*p* = 0.047).

**Conclusions:**

Ga-SPECT/CT using quantitative parameters, namely LBR and TLU, had better diagnostic and prognostic performances for patients with LLOM compared to conventional imaging. The results suggest that Ga-SPECT/CT is a good alternative for diagnosing LLOM in countries where FDG-PET/CT is not commonly available.

## Introduction

Normal bone tissue has high resistance to infection; however, some incidents can cause infection, such as large-volume inoculations, trauma, and the presence of foreign bodies, and the term “osteomyelitis” refers to infection of the bone marrow (Lew and Waldvogel 1997, 2004). In the past 30 years, the incidence of osteomyelitis has nearly tripled among older adults, primarily caused by a drastic increase in diabetes mellitus (Kremers et al. 2014). Osteomyelitis is a difficult-to-diagnose refractory disease characterized by infection and inflammation causing progressive destruction and new bone deposition (Lipsky et al. 2012; Hingorani et al. 2016). Osteomyelitis significantly impacts quality of life and can be fatal (Pedras et al. 2020). Early diagnosis and treatment are crucial for a favorable prognosis (Garcia Del Pozo et al. 2018).

Various imaging modalities have been used to diagnose osteomyelitis. While X-ray, computed tomography (CT), and magnetic resonance imaging (MRI) are widely used for initial diagnosis due to their accessibility (Beaman et al. 2017; Lipsky et al. 2019), their results have limited resolution, which affects diagnostic accuracy (Termaat et al. 2005; Schwegler et al. 2008; Lauri et al. 2017). Nuclear imaging adds another dimension to the diagnosis of LLOM (Beaman et al. 2017). Although nuclear imaging has nearly the same diagnostic accuracy as MRI, nuclear imaging reveals functional abnormalities, such as inflammation activity (Termaat et al. 2005; Becker 1999).

Clinical nuclear imaging uses three main types of tracers for detecting inflammation and infection: ^67^ Ga-citrate (Ga), ^99m^Tc-WBC, and ^18^F-Fluorodeoxyglucose (FDG). The accessibility of tracers varies by location and clinical setting. In several regions, such as Japan, Canada (Ontario), and Australia, ^99m^Tc-WBC and ^18^F-FDG have not yet received general approval for clinical application on musculoskeletal infection, leaving Ga as the standard method (Becker 1999; Ontario Ministry of Health 2015; Government of Western Australia 2019; Department of Health and Aged Care 2022). Ga injected into the bloodstream accumulates in LLOM lesions through increased inflammatory cell uptake and increased receptor density (Tsan 1985; Hoffer et al. 1977). Ga-scintigraphy includes planar and single-photon emission computed tomography combined with CT (SPECT/CT) imaging; however, the spatial resolution and contrast resolution of planar images are low, making it difficult to differentiate LLOM from soft tissue infections, such as cellulitis, resulting in weak or moderate diagnostic performance (Love and Palestro 2016; Delcourt et al. 2005).

SPECT/CT, which simultaneously acquires functional (scintigraphic uptake) and anatomical (low-dose X-ray CT) definition (Horger et al. 2003; Bar-Shalom et al. 2005; Govaert et al. 2017), has resulted in significant improvements to diagnostic confidence (Bar-Shalom et al. 2005).

^18^F-FDG positron emission computed tomography combined with CT (PET/CT) utilizing quantitative analyses with standardized uptake value (SUV) has been widespread globally as well as in some clinical applications in Japan (Miwa et al. 2018); however, PET/CT is generally not approved for LLOM diagnosis in Japan (Nihon Medi-Physics Co. and Ltd. 2022). Furthermore, quantitative analysis software, such as GI-BONE (AZE Co., Ltd, Kawasaki, Japan), has recently been developed to enable quantitative analyses, including using SUV, with SPECT/CT data (Ogura et al. 2019; Hata et al. 2020). However, quantitative analyses using SPECT/CT data have not been applied to evaluate inflammatory activity in LLOM to date.

This study aimed to evaluate the diagnostic accuracy and prognostic value of quantitative Ga-SPECT/CT for patients with LLOM.

## Materials and methods

### Study design

#### Patient selection

**Fig. 1:**
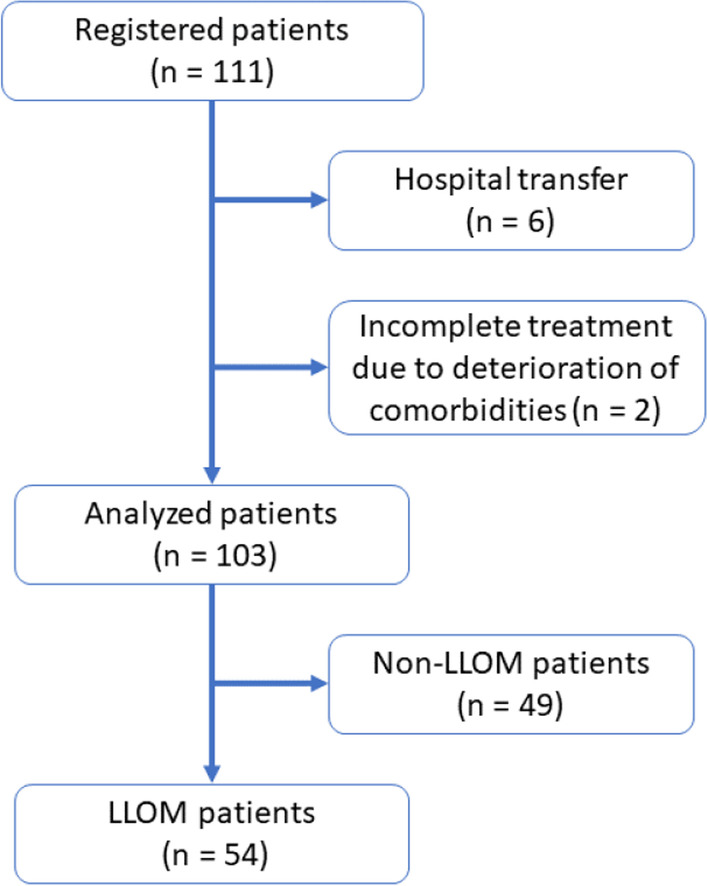
Patient selection flowchart

This study examined 111 consecutive patients suspected of LLOM, because of clinical symptoms including persistent bone pain and findings of infection in blood tests, who underwent Ga-scintigraphy with quantification as part of initial diagnosis between April 2012 and October 2016. Patients who did not complete the required observation were excluded: Six patients were excluded due to incomplete observation resulting from hospital transfer, and two patients were excluded due to incomplete treatment resulting from the exacerbation of comorbidities. Consequently, 103 patients (76 men and 27 women, 67 [55–74] years) were analyzed, as shown in Fig. 1.

#### Ga-scintigraphy imaging procedures

All patients underwent Ga-scintigraphy. SPECT/CT images were obtained 48 h after the injection of 148 MBq of Ga. In addition, planar images were simultaneously obtained for 38 patients. Acquisitions were performed using a SPECT/CT system that contains a dual-head gamma camera with a two-row multi-section CT scanner, Symbia T2 (Siemens Healthcare Japan, Tokyo, Japan). SPECT images were acquired over 15 min per bed position (30 projections over an orbit of 180°, 6° per step, and 30 s per projection). Acquisition range was limited to 2 bed positions from the toes, including the entirety of the lower legs and feet. An MEGP collimator was used for acquisitions with a matrix size of 128 × 128 pixels. SPECT images were reconstructed using an iterative image reconstruction algorithm, Flash3D. Reconstruction parameters for the number of subsets and iterations were 6 and 8, respectively. Non-contrast-enhanced CT scans (tube voltage, 110 kVp; tube current–time product, 10–40 mA; detector configuration, 2 × 4 mm; matrix, 512 × 512 pixels; reconstruction thickness, 5 mm for entire leg and 3 mm for foot) were also performed to obtain morphological data. CT attenuation correction was used to create SPECT/CT images.

Definitive clinical diagnoses were established by the primary physicians using a combination of physical examinations, medical tests, including diagnostic imaging, pathological examinations, and therapeutic diagnosis. The patients received a visual examination of the affected legs, checking for swelling, amount and type of fever, as well as taking blood tests, CT, Ga-scintigraphy, and pathological examinations by biopsy or minor amputation. Patients diagnosed as positive were considered positive for LLOM (LLOM group), while patients diagnosed as negative were considered negative for LLOM (non-LLOM group).

In addition, cellulitis (CE) can interfere with diagnosis, as it may be difficult to distinguish it from LLOM. Furthermore, the presence of both LLOM and CE is not uncommon (Klein et al. 2020). Therefore, patients were additionally classified based on definitive clinical diagnoses of CE established by the primary physicians using the same methods as for LLOM. Four groups were identified: LLOM and CE positive (LLOM-CE), LLOM positive and CE negative (LLOM only), LLOM negative and CE positive (CE only), and LLOM negative and CE negative (LLOM-CE negative).

### Data analysis

Planar, SPECT, and CT images were independently assessed visually and quantitatively by two radiologists, who are certified diagnostic imaging specialists and certified nuclear medicine specialists, in order to identify LLOM. Planar images were visually analyzed to compare lesion accumulation with background accumulation in the unaffected side leg. Lesions inside bone marrow with higher accumulation than background on planar and SPECT images were classified as positive, while those with lower and similar accumulation were classified as negative for LLOM.

Lesions on SPECT and SPECT/CT images were compared to unaffected muscle tissue, and lesions with higher accumulation to background were classified as positive, while those with lower and similar accumulation were classified as negative for LLOM. Osteolytic and sclerotic lesions on CT images were classified as positive for LLOM, and anatomical data obtained from CT images were utilized to identify the precise location of lesions in SPECT/CT images. Following standard procedures, cases that were identified as positive on CT images and planar or SPECT images were considered SPECT/CT positive for LLOM as presented in Table 1.

**Table 1:** Procedure to identify LLOM positive cases using visual assessments

Diagnosis	Modality	Image findings
LLOM	CT	Osteolytic and sclerotic lesions
	Planar imaging	Higher accumulation than background
	SPECT	Higher accumulation than background muscle tissue
	SPECT/CT	Osteolytic and sclerotic lesions with higher accumulation than background muscle tissue
CE	CT	Increased attenuation lesions in subdermal soft tissue
	Planar imaging	Higher accumulation in subdermal soft tissue than background
	SPECT	Higher accumulation in subdermal soft tissue than background
	SPECT/CT	Increased attenuation lesions inside subdermal soft tissue with higher accumulation than background
LLOM + CE	CT	Osteolytic and sclerotic lesions in bone marrow, and increased attenuation lesions in subdermal soft tissue
	Planar imaging	Higher accumulation in both subdermal soft tissue and bone than background
	SPECT	Higher accumulation in both subdermal soft tissue and bone than background
	SPECT/CT	Osteolytic and sclerotic lesions in bone marrow, and increased attenuation lesions in subdermal soft tissue, with higher accumulation than background

In addition, CE was evaluated for differentiation from LLOM visually using planar, SPECT, CT, and SPECT/CT images. Lesions inside the subcutaneous soft tissue with higher accumulation than background on planar and SPECT images were classified as positive, while those with lower and similar accumulation were classified as negative for CE. Increased attenuation value on CT images was classified as positive for CE, and anatomical data obtained from CT images were utilized to identify the precise location of lesions in SPECT/CT images.

Quantitative analyses were performed on lesions in bone marrow suspected to be LLOM-related using SPECT/CT data. Lesion-to-background ratio (LBR) was calculated by dividing maximal count in each lesion accumulation by the mean count of accumulation in the bone marrow of both distal femurs. Furthermore, standardized uptake values (SUV) and total lesion uptake (TLU) were calculated using the quantitative analysis software, GI-BONE. The function of this software is to calculate the Becquerel calibration factor (BCF), and a numeric factor is used to convert a pixel value into the radioactivity density scale similar to PET, using a cylindrical phantom filled with a uniform solution. Using BCF, clinical SPECT images can be converted from a pixel value to an image with a radioactivity density similar to PET, allowing for quantitative analysis. Volume of interest (VOI) threshold was set at 50% of peak value, and maximum SUV (SUVmax) and TLU were calculated. LBR, SUVmax, and TLU cutoff values were determined using a receiver operation curve (ROC) analysis based on definitive diagnoses by primary physicians described above.

Diagnostic values, prognostic values, and visual and quantitative results were compared between the groups.

### Evaluation of prognosis

All patients were observed for five years after their initial Ga-scintigraphy for the occurrence of major adverse events (MAE), which were defined as recurrence of LLOM, major amputation, or mortality caused by the infectious process (apart from myocardial infections). The endpoint for this study was defined as either the occurrence of MAE or end of the observation period. The correlation between the occurrence of MAE and various clinical parameters, including age, the results of blood tests, risk factors, and comorbidities, was also analyzed.

### Statistical analyses

Normally distributed continuous variables were expressed as means ± SD and non-normally distributed variables as medians with 25th and 75th percentiles. Categorical variables were presented as percentages and counts. Non-normally distributed continuous variables, such as age, serum CRP levels, and LBR, were compared using the Mann–Whitney U test.

Categorical variables were compared using Fisher’s exact probability test for bivariate data. In order to examine the correlation with future occurrence of MAE, all variables were checked using a univariate Cox regression analysis. Variables with *p* < 0.05 were considered statistically significant, and the stepwise method was used to select variables for analysis. Multivariate Cox regression was performed on the top four significant variables to identify independently associated factors.

All statistical analyses were performed using StatMate IV software version 4.01 (Advanced Technology for Medicine and Science, Tokyo, Japan) and BellCurve for Excel software version 2.13 (Social Survey Research Information, Tokyo, Japan).

## Results

### Clinical characteristics

A total of 103 patients (67 [55–74] years, 76 men and 27 women) underwent lower-limb Ga-scintigraphy with quantitative SPECT/CT. Planar images were obtained for 38 patients (68 [58–73] years, 29 men and 9 women). Patient characteristics, including medical histories, comorbidities, and blood examination results, are presented in Table 2. Initial treatment for these patients had already been completed, including the administration of antibiotics.

**Table 2:** Patient characteristics

Number of patients	103
Age (years)	67 (55–74)
Male (%)	76 (74%)
Blood exam	
WBC count (/μl)	6200 (5150–7750)
CRP (mg/l)	1.12 (0.28–3.58)
Risk factor	
Diabetes mellitus (%)	77 (75%)
Peripheral artery disease (%)	64 (62%)
Cellulitis (%)	81 (79%)
Comorbidity	
Hypertension (%)	52 (50%)
Chronic kidney disease (%)	49 (48%)
Coronary artery disease (%)	24 (23%)

*WBC* white blood cell; *CRP *C-reactive protein

Of the total, 54 patients were clinically diagnosed as having LLOM (LLOM group), while 49 were clinically diagnosed as not having LLOM (non-LLOM group). Table 3 shows patient characteristics for the two groups. Out of 54 patients in the LLOM group, 49 were clinically identified as having CE (LLOM-CE group) and 5 patients were clinically identified as having only LLOM (LLOM-only group). In addition, 32 patients were clinically diagnosed with CE only (CE-only group) and 17 patients were clinically diagnosed as negative for both LLOM and CE (negative group) (Table 4).

**Table 3:** Comparison of clinical profiles between LLOM and non-LLOM groups

	LLOM (*n* = 54)	Non-LLOM (*n* = 49)	P value
Age (years)	68 (61–75)	64 (55–74)	0.037
Male (%)	37 (69%)	39 (80%)	0.263
Blood exam			
WBC count (/μl)	6250 (5100–7575)	6200 (5400–8000)	0.731
CRP (mg/l)	1.12 (0.24–3.37)	1.62 (0.28–3.72)	0.907
Risk factor/comorbidity			
Diabetes mellitus (%)	37 (69%)	40 (82%)	0.173
Peripheral artery disease (%)	31 (57%)	33 (67%)	0.317
Cellulitis (%)	49 (91%)	32 (65%)	0.003
Hypertension (%)	27 (50%)	25 (51%)	0.925
Chronic kidney disease (%)	21 (39%)	28 (57%)	0.098
Coronary artery disease (%)	10 (19%)	14 (29%)	0.331
Imaging findings			
Positive in planar imaging	12 (71%; n = 17)	14 (67%; n = 21)	0.796
Positive in SPECT/CT	44 (81%)	4 (8%)	< 0.001
LBR	12.23 (7.38–17.94)	1.00 (1.00–1.47)	< 0.001
SUVmax	4.85 (3.45–8.31)	1.34 (1.14–1.62)	< 0.001
TLU	68.77 (22.90–96.63)	8.63 (1.15–2.33)	< 0.001

*LLOM *lower-limb osteomyelitis; *SPECT/CT* single-photon emission computed tomography/computed tomography; *SUV *standardized uptake value;* LBR *lesion-to-background ratio; and *TLU* total lesion uptake

**Table 4:** Results based on the presence of LLOM and CE

	LLOM-CE (*n* = 49)	LLOM only (*n* = 5)	CE only (*n* = 32)	LLOM-CE negative (*n* = 17)
Visual assessment				
CT positive	34 (69%)	3 (60%)	3 (9%)	1 (6%)
SPECT positive	39 (80%)	4 (80%)	26 (81%)	6 (35%)
Quantitative assessment				
LBR	14.86 (8.91–17.40)	9.13 (5.40–8.87)	2.24 (1.00–1.01)	1.86 (1.00–1.01)
SUVmax	6.36 (3.45–8.35)	4.88 (2.02–4.87)	1.56 (1.14–1.58)	1.58 (1.14–1.49)
TLU	69.80 (22.60–96.99)	58.66 (35.02–76.34)	8.51 (1.31–2.36)	8.87 (1.09–2.30)

*CE* cellulitis

#### Visual assessment using Ga-scintigraphy

Based on a visual assessment using planar images, 12 patients (71%) from the LLOM group and 14 patients (67%) from the non-LLOM group were rated as positive for LLOM. SPECT images identified 43 patients (79%) from the LLOM group and 32 patients (65%) from the non-LLOM group as positive for LLOM. CT images categorized 37 patients (69%) from the LLOM group and 4 patients (8%) from the non-LLOM group as positive for LLOM. SPECT/CT images identified 44 patients (81%) from the LLOM group and 4 patients (8%) from the non-LLOM group as positive for LLOM.

Out of the LLOM-CE group, 45 patients (92%), 34 patients (69%), and 39 patients (80%) were rated as positive for LLOM based on planar, CT, and SPECT images, respectively (Table 4). Out of the LLOM-only group, 4 patients (80%), 3 patients (60%), and 4 patients (80%) were rated as positive for LLOM based on planar, CT, and SPECT images, respectively. Out of the CE-only group, 32 patients (100%), 3 patients (9%), and 26 patients (81%) were rated as positive for LLOM based on planar, CT, and SPECT images, respectively. Out of the negative group, 8 patients (47%), 1 patient (6%), and 6 patients (35%) were rated as positive for LLOM based on planar, CT, and SPECT images, respectively.

#### Quantitative assessment using Ga-scintigraphy

Based on clinical diagnosis, LBR, SUVmax, and TLU for the LLOM group were 12.23 (7.38–17.94), 4.85 (3.45–8.31), and 68.77 (22.90–96.63), respectively, and 1.00 (1.00–1.47), 1.34 (1.14–1.62), and 8.63 (1.15–2.33), respectively, for the non-LLOM group (Table 3). The cutoff values for diagnosing LLOM were 1.99 for LBR, 1.74 for SUVmax, and 7.29 for TLU.

The LBR, SUVmax, and TLU in the LLOM-CE group were 14.86 (8.91–17.40), 6.36 (3.45–8.35), and 69.80 (22.60–96.99), respectively. The LBR, SUVmax, and TLU in the LLOM-only group were 9.13 (5.40–8.87), 4.88 (2.02–4.87), and 58.66 (35.02–76.34), respectively. The LBR, SUVmax, and TLU in the CE-only group were 2.24 (1.00–1.01), 1.56 (1.14–1.58), and 8.51 (1.31–2.36), respectively. The LBR, SUVmax, and TLU in the negative group were 1.86 (1.00–1.01), 1.58 (1.14–1.49), and 8.87 (1.09–2.30), respectively. The results demonstrated statistically significant differences in LBR, SUVmax, and TLU between the LLOM-CE and CE-only groups (*p* < 0.001 for all three quantitative parameters).

### Accuracy of imaging methods

As shown in Table 5, the sensitivity and specificity of the planar images were 71% and 33%, respectively. The sensitivity and specificity of the SPECT images were 80% and 35%, respectively. The sensitivity and specificity of the CT images were 69% and 92%, respectively. The sensitivity and specificity of SPECT/CT without quantitative analysis were 81% and 92%, respectively.

**Table 5:** Diagnostic accuracy of imaging modalities

	Sensitivity (%)	Specificity (%)	Accuracy (%)
Visual assessment			
Planar imaging	71	33	50
SPECT imaging	80	35	58
CT imaging	69	92	80
SPECT/CT imaging	81	92	86
Quantitative assessment			
LBR	91	96	94
SUVmax	89	94	92
TLU	91	92	92

The sensitivity and specificity of SPECT/CT with LBR were 91% and 96%, respectively. The sensitivity and specificity of SPECT/CT with SUVmax were 89% and 94%, respectively. The sensitivity and specificity of SPECT/CT with TLU were 91% and 92%, respectively. The areas under the ROC curves for the presence of LLOM were 0.957 using LBR, 0.921 using SUVmax, and 0.926 using TLU.

### Patient prognoses

**Fig. 2:**
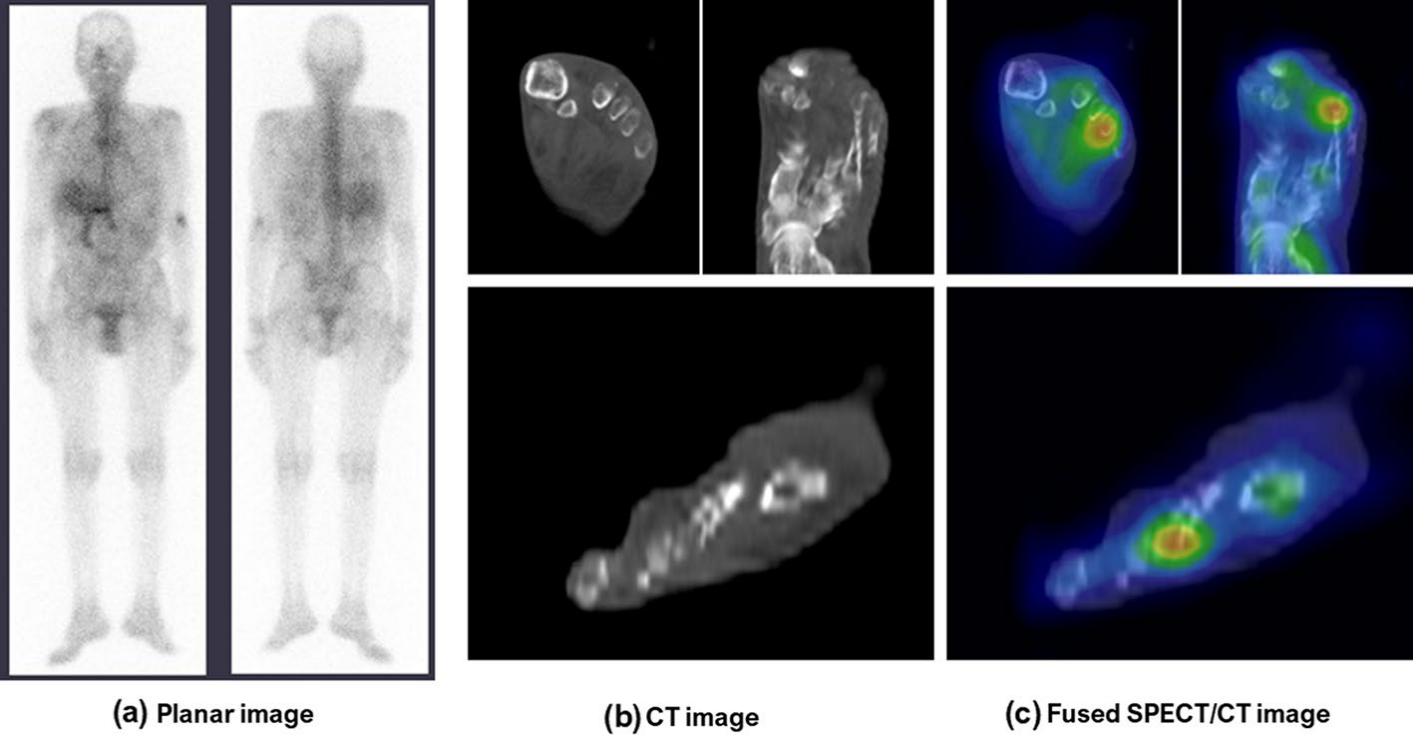
These images represent a 68-year-old man who developed a fever and increased inflammatory markers after treatment for leg trauma, with dyslipidemia, stable angina, and atherosclerosis obliterans as comorbidities. **a** shows Ga-scintigraphy, whole-body, planar images indicating a defect in the left toes with no clear signs of accumulation. **b** shows CT images indicating a resection of the left toes and cellulitis near the left 4th distal phalanx with irregular bone destruction, and increased attenuation lesions in the subcutaneous soft tissue. **c** shows fused SPECT/CT images indicating distinct accumulation in the left 4th distal metatarsal and proximal phalanx with low LBR (5.40), SUVmax (3.25), and TLU (35.02). Recovery from fever and inflammation was smooth, not requiring surgical treatment, but localized pain remained for a few months. The clinical diagnoses of CE and LLOM were established by the primary physicians according to the clinical progress and outcomes. This patient had no MAE within the 3-year observation period

**Fig. 3:**
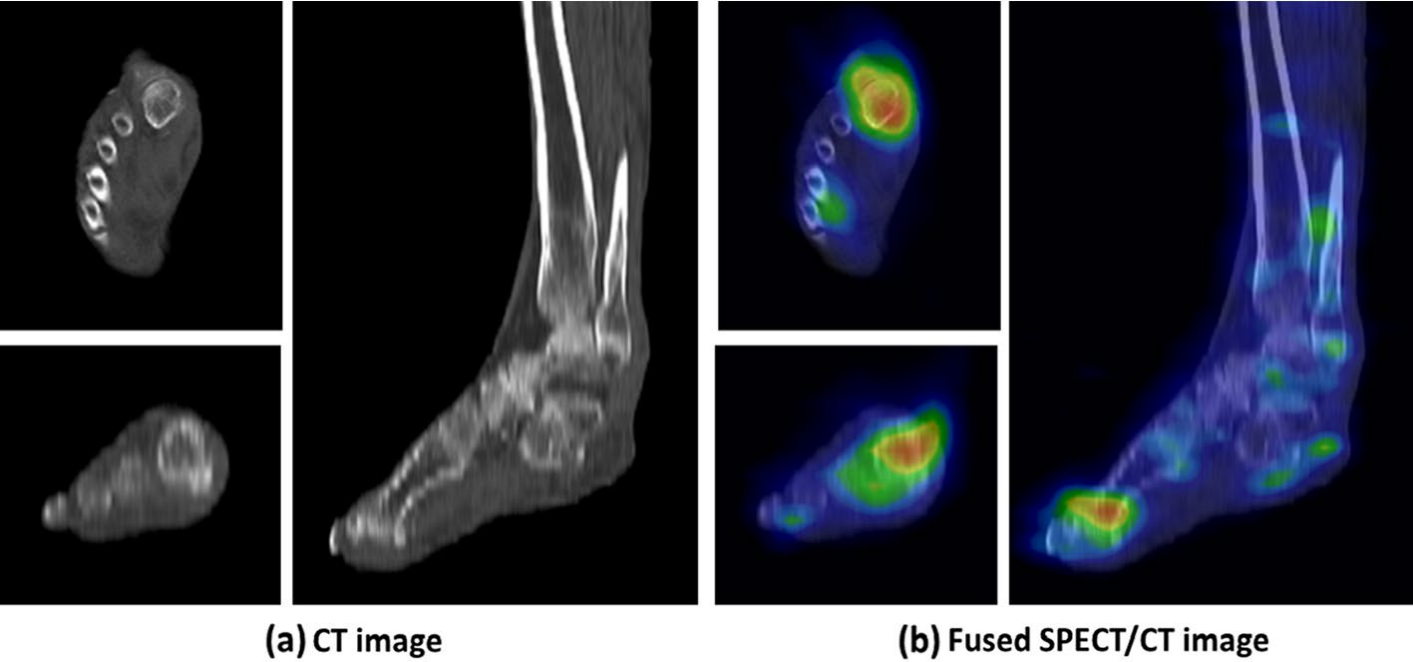
These images represent a 68-year-old man with diabetic gangrene, hypertension, and atherosclerosis obliterans as comorbidities. **a** shows CT images indicating increased subcutaneous density and accumulation indicative of cellulitis near the right 1st proximal phalanx, and metatarsal bone with irregular bone destruction, and increased attenuation lesions in the subcutaneous tissue. **b** shows fused SPECT/CT images indicating distinct accumulation in the subcutaneous tissue, right 1st proximal phalanx and metatarsal bone with high LBR (12.00) and TLU (133.76) and low SUVmax (3.45). The primary physicians diagnosed this patient with CE. The final diagnosis of LLOM was confirmed by the pathologist. The findings indicated sequestrum. Thirty-nine days after the scanning, the patient experienced a fatal event due to sepsis

MAE occurred in 23 patients with LLOM (43%): 19 cases (83%) of major leg amputation, 2 cases (9%) of recurrence of osteomyelitis, and 2 cases (9%) of fatal events. The area under the ROC curve for MAE occurrences was 0.680 for TLU, and the cutoff values for prognosis prediction were 38.35 for TLU. The prevalence of diabetes mellitus and chronic kidney disease as well as WBC count, LBR, and TLU was statistically significantly higher among patients who experienced an MAE (Table 6). The results of the Cox proportional hazards regression analyses are presented in Table 7. The univariate analysis revealed significant correlations for WBC count (*p* = 0.002), diabetes mellitus (*p* = 0.012), TLU (*p* = 0.020), LBR (*p* = 0.030), and chronic kidney disease (*p* = 0.049). A multivariate analysis was performed for the top four parameters and demonstrated a statistically significant positive correlation between WBC count and MAE (*p* = 0.003) as well as TLU and MAE (*p* = 0.047), while LBR showed no statistical significance (*p* = 0.175) (Figs. 2, 3).

**Table 6:** Clinical profiles of patients with LLOM divided by MAE occurrence

	MAE (*n* = 23)	No MAE (*n* = 31)	*P* value
Age (years)	66 (58–69)	68 (61–76)	0.593
Male (%)	16 (70%)	21 (68%)	0.887
Blood exam			
WBC count (/μl)	6800 (5900–8800)	5600 (4800–6550)	0.008
CRP (mg/l)	2.66 (0.30–3.88)	0.79 (0.26–2.41)	0.070
Risk factor/comorbidity			
Diabetes mellitus (%)	21 (91%)	16 (52%)	0.001
Peripheral artery disease (%)	14 (61%)	17 (55%)	0.658
Cellulitis (%)	22 (96%)	27 (87%)	0.284
Hypertension (%)	13 (57%)	14 (45%)	0.409
Chronic kidney disease (%)	12 (52%)	9 (29%)	0.084
Coronary artery disease (%)	6 (26%)	7 (23%)	0.766
Imaging findings			
LBR	18.39 (9.88–17.40)	11.31 (1.00–17.11)	0.017
SUVmax	6.75 (3.45–12.87)	5.83 (2.97–7.78)	0.479
TLU	89.83 (48.00–136.84)	35.02 (19.28–78.53)	0.025
Event-free survival (days)	19 (5.5–57)	NA	NA

*MAE* major adverse event

**Table 7:** Univariate and multivariate Cox regression for MAE occurrence

	Univariate			Multivariate		
	HR	95% CI	P value	HR	95% CI	*P* value
Age	0.986	0.955–1.018	0.399			
Male	1.010	0.415–2.455	0.983			
Blood exam						
WBC count	1.000	1.001–1.001	0.002	1.000	1.000–1.001	0.003
CRP	1.075	0.988–1.169	0.093			
Risk factor/comorbidity						
Diabetes mellitus	6.448	1.508–27.577	0.012	4.081	0.921–18.071	0.064
Peripheral artery disease	1.193	0.516–2.759	0.680			
Cellulitis	2.717	0.366–20.166	0.329			
Hypertension	1.391	0.610–3.173	0.433			
Chronic kidney disease	2.283	1.004–5.191	0.049			
Coronary artery disease	1.182	0.465–3.005	0.725			
Imaging findings						
LBR	1.041	1.004–1.080	0.030	1.030	0.987–1.075	0.175
SUVmax	1.047	0.956–1.146	0.325			
TLU	1.008	1.001–1.016	0.020	1.006	1.000–1.013	0.047

*HR* hazard ratio; *CI *confidential interval

## Discussion

This study evaluated the diagnostic accuracy and prognostic value of quantitative Ga-SPECT/CT for patients with LLOM by comparing it with other methods and clinical diagnoses.

### Comparison of Ga-SPECT/CT and other imaging modalities

An accurate diagnosis of LLOM is crucial for a favorable outcome. However, providing an accurate diagnosis remains a challenge for imaging modalities (Berendt et al. 2008). This study demonstrated that diagnoses with Ga-SPECT/CT using LBR achieved a diagnostic sensitivity and specificity of 91% and 96%, respectively. These results are an improvement over previous attempts lacking quantitative evaluation, which achieved a diagnostic sensitivity and specificity of 88% and 94%, respectively (Aslangul et al. 2013). On the other hand, diagnoses using SUVmax achieved a diagnostic sensitivity and specificity of 89% and 94%, respectively, showing no superiority over previous studies that only used visual evaluation.

SPECT was superior to CT in sensitivity (80% and 69%, respectively), while CT was superior to SPECT in specificity (92% and 35%, respectively). However, SPECT/CT was superior to both SPECT and CT in both dimensions, which may be due to the improvement of the contrast resolution of SPECT images through CT attenuation correction (Seo et al. 2005). In addition, the synergistic effect of the fusion of SPECT and CT combines anatomical data obtained from CT with functional data obtained from SPECT (Bar-Shalom et al. 2005).

FDG-PET/CT has been found to have a sensitivity and specificity for detecting LLOM of 89% and 92%, respectively (Lauri et al. 2017), which is inferior to the performance of Ga-SPECT/CT with LBR observed in the present study. However, analyses utilizing FDG-PET/CT have not incorporated LBR to date. As such, future research on the possibility of using LBR with FDG-PET/CT may shed more light on this issue.

### Comparison of quantitative evaluation methods

The results demonstrated the diagnostic significance of LBR, SUVmax, and TLU for LLOM; however, while TLU was positively correlated with prognosis, LBR and SUVmax were not statistically significant. This was likely due to the fact that SUVmax was based on a calculated distribution value, resulting in data related to inflammation other than LLOM (Takaki et al. 2020). On the other hand, LBR was based on a comparison of the affected tissue and unaffected tissue, avoiding confusion with other sites. Unlike SUV and TLU, LBR-based calculations were similar to a radiologists’ visual interpretation. However, as measurement location, including background, was determined manually, LBR-based calculations could be vulnerable to error. On the other hand, TLU was a more objective measure and more accurate assessment of local inflammatory activity.

### Prognostic value of Ga-SPECT/CT with quantitative parameters

As mentioned, Ga-SPECT/CT is generally not the preferred modality for LLOM in most countries; therefore, its prognostic value has not been investigated to date. The use of Ga-SPECT/CT in the literature has been limited. For instance, Aslangul et al. reported that combined diagnosis with Ga-SPECT/CT and percutaneous bone puncture improved the 1-year outcome of patients with LLOM (4 improved and 15 cured out of 55 patients) (Aslangul et al. 2013). However, the present study revealed the efficacy of Ga-SPECT/CT as a prognostic tool. The multivariate analysis revealed TLU to be an independent prognostic factor (*p* = 0.047). The results demonstrated that prognosis was significantly poorer in patients with high TLU than those with low TLU.

Similarly, the prognostic value of FDG-PET/CT for LLOM has not been determined. However, FDG-PET/CT has been reported to improve LLOM diagnosis and therapeutic monitoring and effects (Chatziioannou et al. 2015). Furthermore, surgery based on FDG-PET/CT images using SUV cutoff values of 2.00–8.00 has a higher potential for procedural success (Takaki et al. 2020). This suggests that FDG-PET/CT is likely to have a good prognostic value.

However, as mentioned, FDG-PET/CT generally cannot be used in certain regions due to technical and insurance limitations. Therefore, Ga-SPECT/CT presents the best available method with a potential for high prognostic value. Providing an accurate prognosis would allow early intervention and mitigate some of burdens. This study indicated that quantitative assessment is more precise than visual assessment and enables prognosis stratification. The results provided strong evidence for recommending the utilization of Ga-SPECT/CT for patients with LLOM, at least in countries where FDG-PET/CT is not available or feasible. Future research should investigate this method across Japan and in other countries in order to lend further validity to these results.

### Study limitations

This study had some limitations. First, due to the retrospective design of the study, there may be a bias in case selection caused by the initial focus on the indication for surgery. Moreover, for the same reason, clinical examinations may not have been optimized for LLOM, such as injection-to-scan acquisition times. Future research should conduct multicenter randomized controlled trials in order to eliminate this potential bias. Second, Ga-SPECT/CT was chosen over FDG-PET/CT, as Japan’s national health insurance system only covers Ga-scintigraphy for patients with LLOM. Further research should explore the hypothesis that FDG-PET/CT is likely to have a good prognostic value, as mentioned. Furthermore, the sample size of the present study was relatively small. Future studies should aim to include a wider range of participants.

## Conclusions

This study evaluated inflammatory activity in patients with LLOM using quantitative Ga-SPECT/CT. The results indicated that Ga-SPECT/CT using quantitative parameters, namely LBR, SUVmax, and TLU, had a better diagnostic performance for patients with LLOM compared to planar imaging. In addition, this study found that TLU values were positively correlated with MAE, demonstrating the prognostic assessment potential of Ga-SPECT/CT with TLU, including the ability to stratify the prognosis of patients with LLOM.

The results suggest that Ga-SPECT/CT is a good alternative for diagnosing LLOM in countries where FDG-PET/CT is not commonly available. In addition, the results suggest the possibility of adding new clinical value in predicting prognosis by introducing quantitative analyses at facilities that are already performing Ga-SPECT/CT, without installing any additional hardware. Such facilities could provide additional information from quantitative analyses to physicians that improves treatment strategies. It should be noted that although Ga-SPECT/CT is an acceptable alternative, most physicians agree that FDG-PET/CT is superior (Klein et al. 2020). Therefore, future policies should strive to allow the implementation of FDG-PET/CT for LLOM whenever possible.

## Data Availability

No datasets were generated or analyzed during the current study.
